# Effectiveness of pre-cooling and cooling during play on wheelchair rugby performance

**DOI:** 10.1186/2046-7648-4-S1-A4

**Published:** 2015-09-14

**Authors:** Katy Griggs, George Havenith, Michael Price, Thomas Paulson, Victoria Goosey-Tolfrey

**Affiliations:** 1 Peter Harrison Centre for Disability Sport, Loughborough University, UK; 2 Environmental Ergonomics Research Centre, Loughborough University, UK; 3 Department of Biomolecular and Sports Science, Coventry University, UK

## Introduction

Athletes with tetraplegia (spinal cord injury at the cervical region of the spinal cord) are at a greater risk of heat illness than their able-bodied counterparts, due to the loss of sweating capacity and vasomotor control below the lesion level. Commercially available ice vests worn prior to exercise (pre-cooling) have received considerable interest in the able-bodied athletic population eliciting varying results in performance, and physiological and thermoregulatory responses. However, limited research has been conducted in thermoregulatory impaired athletes with tetraplegia. Anecdotally water spraying the face and torso is commonly used by these athletes during breaks in play, though the effectiveness of this method has not been established. The purpose of this study was to investigate the effects of pre-cooling using an ice vest and the combination of pre-cooling and cooling during play using water sprays on simulated wheelchair rugby performance in athletes with tetraplegia.

## Methods

Eight wheelchair rugby players with tetraplegia (32(7) yrs, 64.0(6.8) kg, $\overset{\cdot }{\text{V}}{\text{O}}_{\text{2peak}}$ 1.35(0.27) mL.kg.min^-1^) completed a 60 min intermittent sprint protocol (ISP) on a wheelchair ergometer in 20.2(0.2) °C and 33.0(3.1)% rh. The ISP represented a wheelchair rugby match (4 quarters) and was based on data obtained from competitive match play by an indoor tracking system. The ISP was conducted on three occasions either with no cooling (NC), pre-cooling with an ice vest (P) or pre-cooling with an ice vest and water sprays between quarters (PW). The ice vest (Artic Heat Products) weighed ~800g when activated, was worn over the top of the participant's playing vest and was applied during a 15 min rest period and subsequent 20 min warm-up. In PW, water (~ 17 °C) was sprayed twice (20 s spray) on the face, fronts of both arms and torso with a water spray (~50 mL per 20 s spray) at the end of each quarter. Gastrointestinal temperature was measured by a telemetry pill (T_gi_), individual and mean skin temperature (T_sk, _Ramanathan method), heart rate, rating of perceived exertion (RPE), thermal sensation and thermal comfort were measured throughout in addition to wheelchair performance.

## Results

At the end of the pre-cooling period, T_gi _was 0.3(0.2) °C, 0.2(0.3) °C and 0.2(0.4) °C higher than at the start for NC, P and PW, respectively (p > 0.05). The reduction in T_sk _over the pre-cooling period was significantly greater in P and PW compared to NC (-0.8(0.6) °C, -1.6(0.8) °C and -1.2(0.5) °C for NC, P and PW, respectively, p < 0.05). The ΔT_gi _over the ISP was significantly lower from the end of quarter 2 to completion of the ISP in PW and P compared to NC (p < 0.05). By the end of the second quarter to completion of the ISP, the reduction in T_sk _was lower in PW compared to P and NC (p < 0.05). Cooling had no effect on heart rate, performance measures, RPE, thermal sensation or thermal comfort during the ISP.

## Discussion

Pre-cooling using an ice vest attenuated T_sk _during the pre-cooling period, and although this reduction was not long lasting ΔT_gi _on completion of the ISP was still lower in P compared to NC. PW lowered thermal strain to a greater degree than P, yet neither condition had a detrimental effect on performance implying cooling did not affect the participant's pushing ability. Participants reported no differences in thermal sensation or comfort between conditions, suggesting they were unable to detect the attenuation in Tsk, potentially due to the small area of sensate skin in individuals with tetraplegia.

## Conclusion

Water spraying between quarters in addition to pre-cooling with an ice vest lowers thermal strain to a greater degree than pre-cooling only, and has no detrimental effect on key parameters of wheelchair rugby performance or thermal perceptions.

